# Lactic Acid Bacteria-Derived Postbiotics as Adjunctive Agents in Breast Cancer Treatment to Boost the Antineoplastic Effect of a Conventional Therapeutic Comprising Tamoxifen and a New Drug Candidate: An Aziridine–Hydrazide Hydrazone Derivative

**DOI:** 10.3390/molecules29102292

**Published:** 2024-05-13

**Authors:** Joanna Wasiak, Pola Głowacka, Agnieszka Pudlarz, Adam M. Pieczonka, Katarzyna Dzitko, Janusz Szemraj, Monika Witusik-Perkowska

**Affiliations:** 1 Department of Medical Biochemistry, Medical University of Lodz, 6/8 Mazowiecka Str., 92-215 Lodz, Poland; joanna.wasiak@umed.lodz.pl (J.W.); pola.glowacka@umed.lodz.pl (P.G.); agnieszka.pudlarz@umed.lodz.pl (A.P.); janusz.szemraj@umed.lodz.pl (J.S.); 2 Department of Organic and Applied Chemistry, Faculty of Chemistry, University of Lodz, Tamka 12 Str., 91-403 Lodz, Poland; adam.pieczonka@chemia.uni.lodz.pl; 3 Department of Molecular Microbiology, Faculty of Biology and Environmental Protection, University of Lodz, Banacha 12/16 Str., 90-237 Lodz, Poland; katarzyna.dzitko@biol.uni.lodz.pl

**Keywords:** lactic acid bacteria, *L. plantarum*, *L. rhamnosus*, postbiotics, breast cancer, tamoxifen, aziridine–hydrazide hydrazones, adjunctive therapy

## Abstract

Breast cancer is associated with high mortality and morbidity rates. As about 20–30% of patients exhibiting ER-positive phenotype are resistant to hormonal treatment with the standard drug tamoxifen, finding new therapies is a necessity. Postbiotics, metabolites, and macromolecules isolated from probiotic bacteria cultures have been proven to have sufficient bioactivity to exert prohealth and anticancer effects, making them viable adjunctive agents for the treatment of various neoplasms, including breast cancer. In the current study, postbiotics derived from *L. plantarum* and *L. rhamnosus* cultures were assessed on an in vitro breast cancer model as potential adjunctive agents to therapy utilizing tamoxifen and a candidate aziridine–hydrazide hydrazone derivative drug. Cell viability and cell death processes, including apoptosis, were analyzed for neoplastic MCF-7 cells treated with postbiotics and synthetic compounds. Cell cycle progression and proliferation were analyzed by PI-based flow cytometry and Ki-67 immunostaining. Postbiotics decreased viability and triggered apoptosis in MCF-7, modestly affecting the cell cycle and showing a lack of negative impact on normal cell viability. Moreover, they enhanced the cytotoxic effect of tamoxifen and the new candidate drug toward MCF-7, accelerating apoptosis and the inhibition of proliferation. This illustrates postbiotics’ potential as natural adjunctive agents supporting anticancer therapy based on synthetic drugs.

## 1. Introduction

Breast cancer is one of the most common cancers among females and is associated with high mortality and morbidity rates. Estrogen receptor (ER)-positive breast cancer represents almost 70% of all cases, for which tamoxifen (TAM) or its derivatives are the most commonly used therapeutic. TAM acts as an antagonist, leading to estrogenic pathway inhibition and influencing cancer cell growth or proliferation. Despite ER presence, about 20–30% of patients demonstrate intrinsic resistance to hormonal treatment; thus, it is important to find new therapeutic solutions [1]. There are indications that combination therapy based on TAM and certain compounds of natural origin improves the responsiveness of ER-positive breast cancer cells to treatment [2]. On the other hand, a growing body of evidence has emphasized the role of microbiota and probiotic supplementation in maintaining health and the prophylaxis of several diseases, including cancer. Interestingly, recent findings have demonstrated that not only the living microbes but also postbiotics—their isolated metabolites or macromolecules—present sufficient bioactivity to influence cellular and physiological processes occurring in the human body. Since postbiotics can exert a beneficial prohealth effect, they can be considered a safe alternative to living probiotic cells. These findings are especially important for food and pharmaceutical industries since maintaining probiotics in their viable form in food products or supplements would pose a major challenge [3,4]. Postbiotics have been proven to be involved in having various healthful effects, including immunomodulation, anti-inflammatory responses, and the maintenance of gut barrier integrity [5]. Recent research has also emphasized the antineoplastic potential of probiotic metabolites targeted selectively to cancer cells, making postbiotics a promising means for supporting cancer therapies. Microbial metabolites can act locally or enter the systemic circulation and exert prohealth effects at distant sites, which makes them an interesting therapeutic option not only for digestive tract cancers but also for neoplasms located at other body sites. Existing data allow us to single out several molecules or metabolites responsible for this effect, e.g., bacteriocins, exopolysaccharides, peptidoglycans, and short-chain fatty acids (SCFAs). However, the mechanisms of their actions are poorly understood and are still being investigated, mainly using various models of colorectal cancer [3,6,7]. Our study was aimed to validate the supportive antineoplastic potential of *Lactobacillus rhamnosus* GG and *Lactiplantibacillus plantarum* 299v as the most investigated lactic acid bacteria (LAB) strains, as they are also commonly utilized in pharmaceutical and food industries [8,9,10]. Using an in vitro breast cancer model, postbiotics derived from the aforementioned LAB strains were assessed as potential adjunctive agents to oncotherapy based on TAM. Additionally, postbiotics’ influence on the anticancer activity of a new candidate compound belonging to the class of aziridine–hydrazide hydrazone derivatives (ARA12) was evaluated. The preparation and antineoplastic activity of the new candidate drug has been previously described by our team against glioblastoma cells [11]. 

## 2. Results

### 2.1. LAB Postbiotics Enhance the Cytotoxic Effect of Classic Tamoxifen Treatment

**Figure 1:**
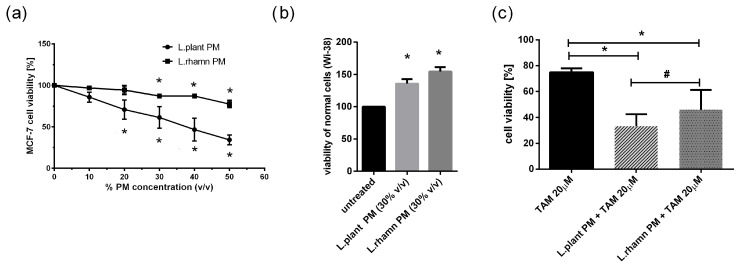
Response of breast cancer cells to LAB-derived postbiotics and their combinations with tamoxifen based on resazurin viability assay: (**a**) survival analysis of MCF-7 after 72 h treatment with a range of PM concentrations obtained from tested LAB strains revealed a significant anticancer effect for 20% (*v*/*v) L. plantarum* PM and for 30% (*v*/*v*) *L. rhamnosus* PM (*); *p* < 0.05; (**b**) *L. plantarum* PM and *L. rhamnosus* PM positively influenced viability of normal fibroblast cells (WI-38) assessed in relation to untreated control (*); *p* < 0.05; (**c**) combinations of TAM (20 µM) with LAB-PM (30% *v*/*v*) exerted a significantly enhanced antineoplastic effect in comparison to tamoxifen alone (*) with stronger inhibitory activity observed for mixture of *L. plantarum* derived PM and TAM (#); *p* < 0.05.

To initially evaluate the anticancer potential of LAB metabolites, the viability of MCF-7 treated with an increasing concentration of LAB-PM was assessed. A significant decrease in cell viability in comparison to the untreated control was detected for 20% *v*/*v* of *L. plantarum* PM and 30% *v*/*v* of *L. rhamnosus* PM after 72 h of treatment (Figure 1a). In addition, the selectivity of the antineoplastic effect was verified on a normal fibroblast cell line (WI-38), showing a positive influence on cell viability. After 72 h of treatment with 30% *v*/*v* *L. planatrum* PM, a 1.35-fold increase in their viability status was detected, while *L. rahmnosus* PM caused a 1.54-fold rise in the viability of WI-38 cells measured by resazurin conversion (Figure 1b). Since 30% *v*/*v* appeared to be an effective concentration of LAB-PM derived from both *L. plantarum* and *L. rhamnosus* causing no harmful effects on normal cells (WI-38), this concentration was selected for further analyses (Figure 1a). Based on the concentration-dependent analysis of MCF-7 cells treated with TAM, TAM 20 µM was used for testing the combination therapy effect. The results demonstrate that LAB metabolites increase the effectiveness of TAM, causing about a 40% decrease in cell viability after *L. plantarum* addition and about a 30% decrease after *L. rhamnosus* application in relation to the viability of cells treated with TAM alone; *p* < 0.05 (Figure 1c).

**Figure 2:**
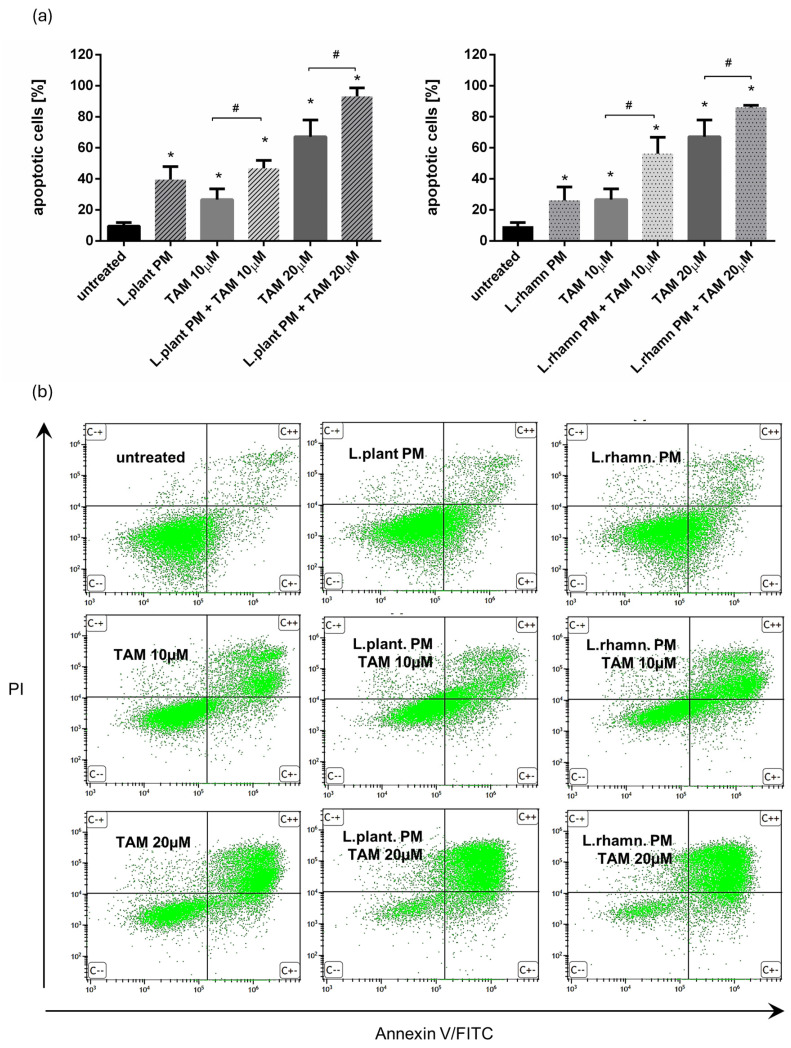
Results of cell viability analysis based on annexin V/PI assay performed for MCF-7 cells treated with LAB-derived PMs and their combinations with TAM: (**a**) quantitative FAC analysis demonstrated prodeath activity for *L. plantarum* PM and *L. rhamnosus* PM, detected as a significant increase in apoptotic cell percentage when compared to untreated control (*); *p* < 0.05. The addition of *L. plantarum* PM or *L. rhamnosus* PM to TAM resulted in the significant enrichment of the apoptotic population in comparison to TAM alone for both tested drug concentrations (#); *p* < 0.05; (**b**) representative FAC results showing proapoptotic activity of LAB-PM improving anticancer potential of TAM.

Further analyses demonstrated that the proapoptotic effect of TAM was enhanced by the addition of postbiotics derived from examined LAB strains, resulting in a significant increase in the percentage of apoptotic cells. Two concentrations of TAM (10 and 20 µM) were tested to expose the influence of LAB-PM on the cell death of MCF-7. FAC-based analysis confirmed the anticancer effect of LAB-derived PM, yielding an output of 39.52 ± 8.42% of apoptotic cells after *L. plantarum* PM and 26.09 ± 8.64% of apoptotic cells after *L. rhamnosus*-derived PM were applied for 72 h. When compared to the effectivity of 10 µM TAM treatment (26.67 ± 6.88% of apoptotic cells), a combination of 10 µM TAM with LAB-derived PM caused about two-fold increase in the percentage of apoptotic cells, enriching the apoptotic cell population to 46.86 ± 5.06% for *L. plantarum*-derived PM addition and 56.26 ± 10.60% when *L. rhamnosus*-derived PM was added. A combination of LAB-derived PMs with higher TAM concentration (20 µM) also resulted in a significant increase in apoptotic cell population—about 20–25% when compared to the effectivity of TAM alone (67.11 ± 10.86%), ultimately yielding 93.13 ± 5.54% for TAM mixed with *L. plantarum* PM and 86.01 ± 1.39% for TAM applied in parallel with *L. rhamnosus* PM (Figure 2). 

**Figure 3:**
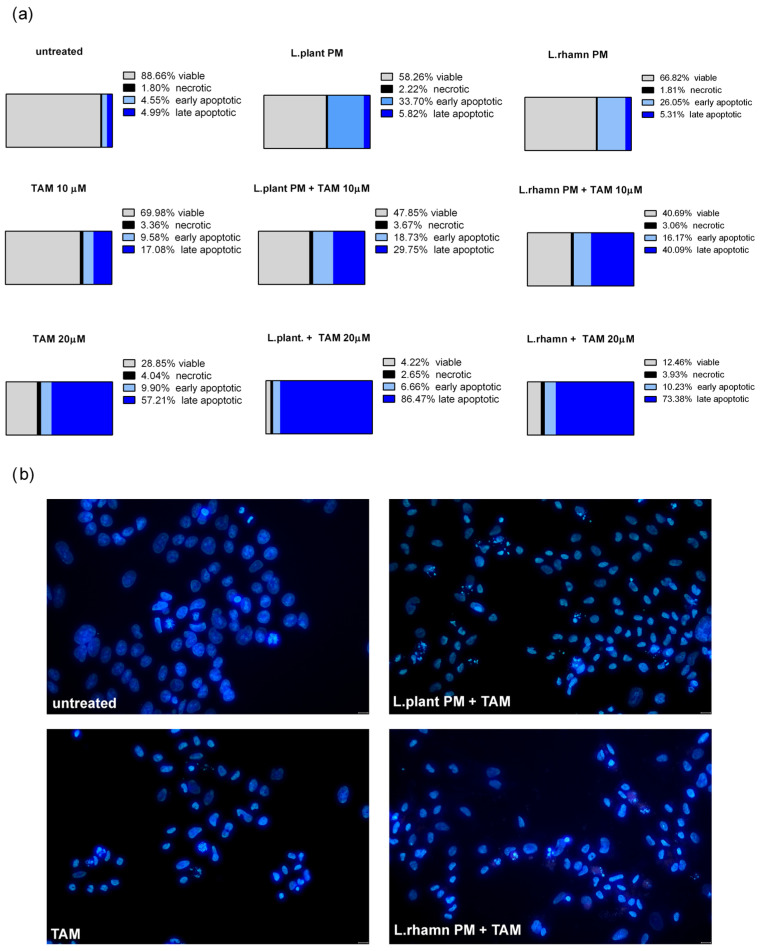
Cell death analysis for MCF-7 cells treated with *L. plantarum*- or *L. rhamnosus*-derived PM and their combinations with TAM: (**a**) quantitative results of annexin V/PI assay (after 72 h) considering 4 types of cell population, namely viable, necrotic, early apoptotic, and late apoptotic, which reveal the acceleration of apoptosis process stimulated by a mixture of TAM and LAB-derived PM, identified as an enrichment of late apoptotic population in comparison to cells treated with LAB-PM alone; (**b**) the advancement of apoptosis initiated by examined modes of treatment is confirmed by the presence of cells demonstrating changes in the nuclear morphology, visualized by DAPI staining after 48 h.

As FAC results demonstrated, postbiotics derived from *L. plantarum* and *L. rhamnosus* have the ability to stimulate apoptosis in MCF-7; however, when LAB-PM was applied as a single factor, the cells detected as apoptotic were found mainly in the early apoptotic phase (33.7% for *L. plantarum* and 66.82% for *L. rhamnosus* PM). TAM with LAB-PM resulted in a significant enrichment of the late apoptotic population in a dose-dependent manner. The addition of LAB-PM to 10 µM TAM resulted in about 5–8-fold increase in the late apoptotic population, while the combination with 20 µM TAM enriched it about 15 times when compared to MCF-7 treated with LAB postbiotics alone (Figure 3a). Although the general proapoptotic activity of *L. plantarum* PM appeared to be comparable to that of the combination with 10 µM TAM (Figure 2), the higher efficiency of the latter is evidenced by the acceleration of apoptosis (Figure 3a). The proapoptotic activity of the combinatory treatment mode was confirmed by the presence of cells presenting features of apoptosis, such as nuclei condensation and fragmentation (Figure 3b).

### 2.2. Antineoplastic Effect of New Candidate Compound ARA12 Is Increased by LAB Postbiotics 

**Figure 4:**
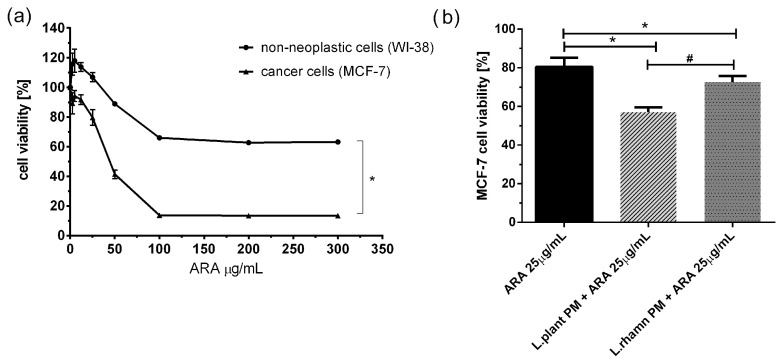
Response of breast cancer cells to new candidate drug ARA12 and its combinations with LAB-derived postbiotics: (**a**) survival analysis of cancer cell line MCF-7 and normal cell line WI-38 revealed selective antineoplastic activity of ARA12, identified as a significantly stronger response of breast cancer cells (*); *p* < 0.05; (**b**) anticancer efficiency of ARA12 is significantly enhanced by addition of LAB-PM (*) with stronger effect detected for combination of candidate drug with *L. plantarum* PM (#); *p* < 0.05.

To examine the adjunctive potential of LAB postbiotics in cancer therapy, the effect of combinatory treatment was also tested for a new candidate compound presenting antineoplastic potential, ARA12, which is a new derivative of aziridine–hydrazide hydrazones [11]. The initial multiwell-plate-based assay showed that ARA12 has selective anticancer activity against breast cancer cells, causing a significant decrease in MCF-7 viability. A concentration of 25 µg/mL was selected for further experiments, and it was found to be not harmful to normal cells but significantly toxic for cancer cells, decreasing the viability of MCF-7 to 80.52 ± 4.74% after 72 h; *p* < 0.05. A mixture of ARA12 with LAB-derived postbiotics resulted in a decrease in cell viability to 57.17 ± 2.45% when combined with *L. plantarum* PM and 72.74 ± 3.02% for cells treated in parallel with *L. rhamnosus* PM. For both combinatory modes of treatment, the effect appeared to be significantly stronger than that detected after a single application of ARA 25 µg/mL; *p* < 0.05 (Figure 4).

**Figure 5:**
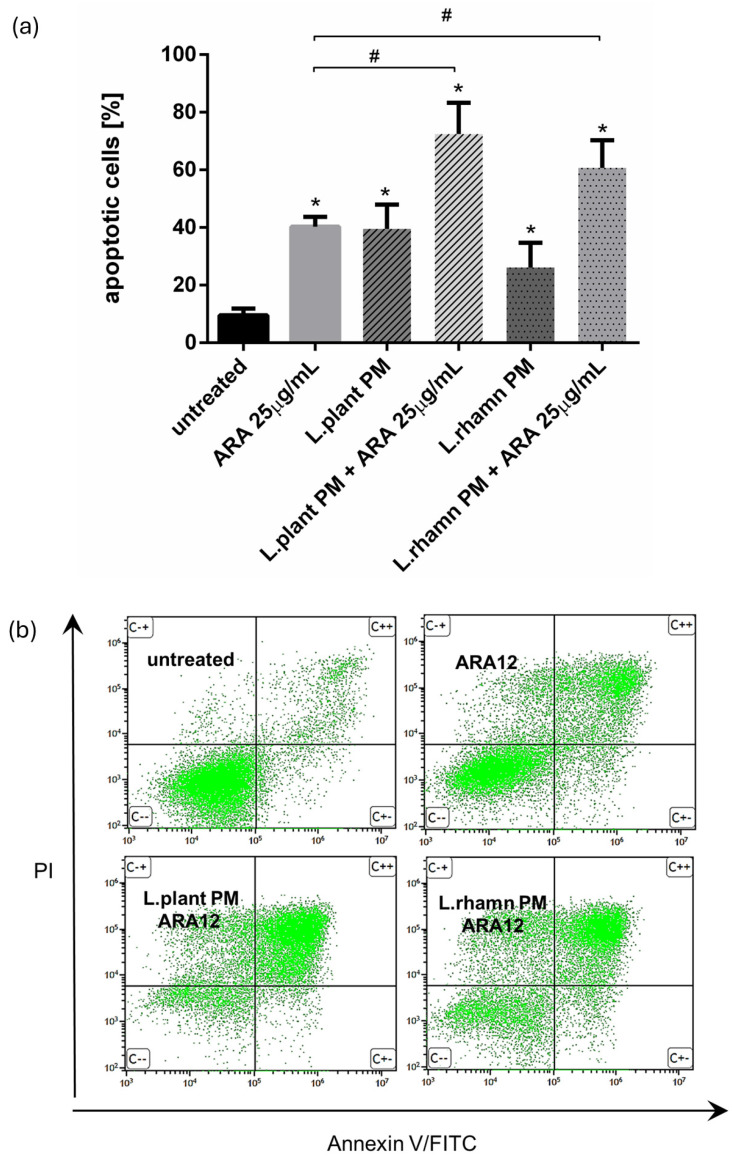
Results of cell viability analysis based on annexin V/PI assay performed for MCF-7 cells treated with LAB-derived PMs and their combinations with ARA12: (**a**) quantitative FAC analysis demonstrated prodeath activity for all of the treatment modes, identified as an enrichment of apoptotic population in comparison to control (*); however, the mixture of ARA12 with *L. plantarum* PM or *L. rhamnosus* PM significantly increased apoptosis efficiency when compared to that detected to synthetic compound (#); *p* < 0.05; (**b**) representative FAC results presenting proapoptotic activity of ARA12 enhanced by LAB-PM.

FAC analysis targeted to cell death detection by annexin V/PI staining confirmed that ARA12 is able to stimulate apoptosis in MCF-7 cells, yielding an output of 40.32 ± 3.36% of apoptotic cells after 72 h of treatment. When a mixture of ARA12 with LAB-PM was applied, the prodeath effect was significantly enhanced by about 20–30%, generating 72.45 ± 10.83% of apoptotic cells after the addition of *L. plantarum* PM and 60.67 ± 9.63% of apoptotic cells when *L. rhamnosus* PM was added; *p* < 0.05 (Figure 5).

**Figure 6:**
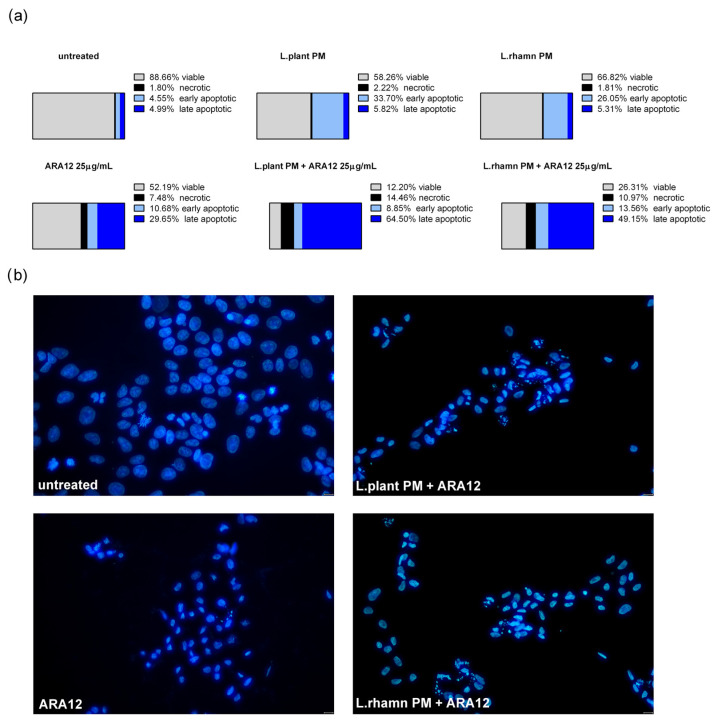
Cell death analysis for MCF-7 cells treated with *L*. *plantarum-* or *L. rhamnosus*-derived PM and their combinations with ARA12: (**a**) the quantitative outcomes of the annexin V/PI assay (after 72 h), assessing 4 distinct cell populations, namely viable, necrotic, early apoptotic, and late apoptotic, indicated an expedited apoptosis process induced by the combinations of ARA12 and LAB-derived PMs, identified as an enrichment of late apoptotic population compared to cells treated with LAB-PMs alone; (**b**) apoptosis induced by examined modes of treatment was confirmed by the observation of apoptotic changes in nuclear morphology, as revealed through DAPI staining after 48 h.

Similar to the results detected for TAM, ARA12 addition to LAB-derived PM led to a significant increase in the late apoptotic population from an average value of about 5% after a single treatment with LAB-derived PM to 64.5% from a combinatory treatment of ARA12 with *L. plantarum* and 49.15% after the application of ARA12 mixture with *L. rhamnosus* PM (Figure 6a). The proapoptotic potential of this treatment approach was also confirmed by the detection of cells presenting specific changes in nuclear morphology visualized with DAPI staining (Figure 6b).

### 2.3. Influence of LAB-Derived Postbiotics on Cell Cycle and Proliferation of Cancer Cells

**Figure 7:**
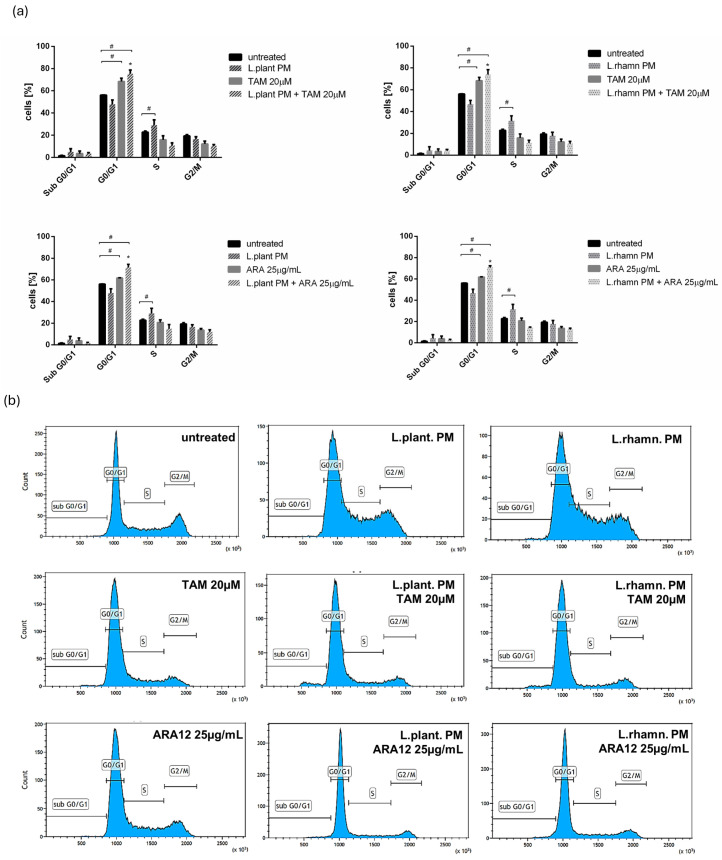
Analysis of cell cycle of breast cancer cells affected by LAB-derived PMs and their combinations with synthetic compounds TAM and ARA12 for 48 h: (**a**) estimation of fractional DNA content by PI incorporation using FACs in MCF-7 treated with *L. plantarum* or *L. rhamnosus* PM shows the influence of postbiotics on cell cycle, identified as a shift to the S phase (#); *p* < 0.05. Combinations of LAB-PMs with synthetic compounds inhibited the cell cycle in the G0/G1 phase (#), significantly enhancing (*) this effect, which was also detected for TAM (20 µM) (#) and ARA12 (25 µg/mL) (#) alone; *p* < 0.05; (**b**) representative results of cell cycle analysis using PI incorporation assay for MCF-7 subjected to various mode of treatments.

**Figure 8:**
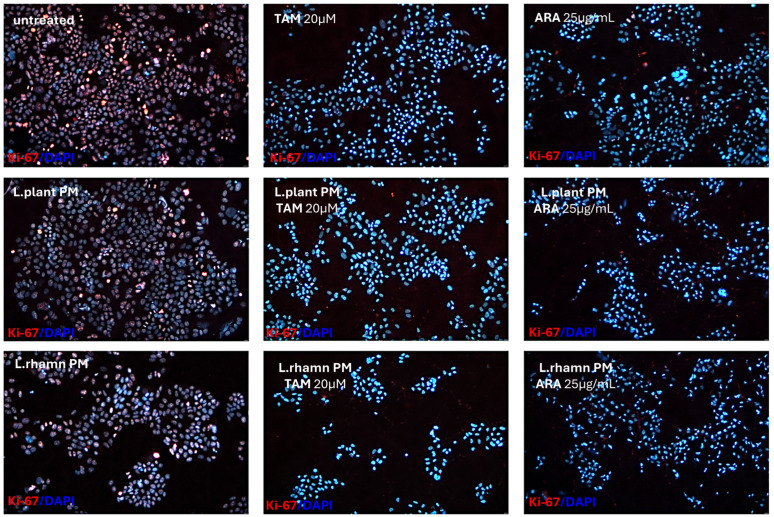
Proliferation status of breast cancer cells treated with LAB-PMs, synthetic compounds, and their combinations. Immunofluorescence analysis of Ki-67, which is considered a proliferation marker, demonstrated the influence of LAB-derived PMs on its expression pattern and the downregulation of its level to an almost undetectable status in cells treated with chemical compounds TAM and ARA12 and their combinations with postbiotics. Quantitative analysis demonstrated that 92.6% of untreated cells were positive for Ki-67, while LAB-PM application resulted in a slight but significant decrease in their number to 84.5% for *L. plantarum* PM-treated cells and 87.6% for the population treated with *L. rhamnosus* PM (*p* < 0.05). Synthetic agents caused a strong reduction in Ki-67 expressing cells to the level of 1.8% after TAM and 3.3% after ARA12 application (*p* < 0.05), while combinatory treatment with postbiotics yielded an output of <1% of cells presenting a weak signal of Ki-67.

The anticancer effect of LAB-derived postbiotics was also investigated in the context of their influence on the proliferation and cell cycle of MCF-7. After the 48-h treatment with *L. plantarum* PM or *L. rhamnosus* PM, a modest effect on the cell cycle was observed, detected mainly as an enrichment in the S population; *p* < 0.05 (Figure 7). The immunofluorescence analysis of proliferation markers demonstrated changes in the Ki-67 expression pattern in LAB-PM-treated MCF-7 in comparison to the untreated control, visualized as a decrease in the number of cells presenting the highest antigen level with the perichromosomal localization typical of mitotic cells, from 7.6% in the control to 3.3–3.7% in cells treated with LAB-PM (*p* < 0.05). However, the majority of cells subjected to LAB-PM were still positive for Ki-67 occurring as small foci—81.2% after *L. planatrum* PM and 83.9% after *L. rhamnosus* PM application (Figure 8). Treatment with synthetic agents TAM and ARA12 resulted in a shift to the G1 phase along with almost no detection of Ki-67 immunofluorescence signal (1.8% and 3.3% of positive cells after TAM and ARA12 application, respectively), while the combination therapy enhanced this effect, causing a significant increase in the G1 population (*p* < 0.05) and the downregulation of Ki-67 expression to undetectable level typical for quiescent cells, which suggests the inhibition of MCF-7 proliferation (Figure 7 and Figure 8).

## 3. Discussion

Interactions between the human microbiome and health have lately emerged as a focal point in scientific research. According to recent findings, there is a connection between gut dysbiosis and a higher probability of breast cancer, as changes in microbial composition have an influence on estrogen metabolic pathways. Intestinal bacteria can produce the beta-glucuronidase enzyme, altering estrogens into their active forms and increasing the availability of intestinal estrogens for resorption in the bloodstream. An imbalance in the gut can lead to an excess or insufficient amount of this enzyme, which disrupts the hormonal equilibrium in the body and may contribute to carcinogenesis [12,13]. Importantly, gut dysbiosis is not the only factor involved in this phenomenon, since several studies have shown that the imbalance of breast microbiome also correlates with breast cancer incidence. Skin and oral bacteria have been observed to enter the breast ducts through the nipple, but some scientific reports suggest that the place of their origin is the mother’s gastrointestinal tract [14,15].

On the other hand, recent advancements highlight the potential of probiotics as promising agents in breast cancer prophylaxis and therapy [16]. Combined with chemotherapy or immunotherapy, probiotics could enhance treatment efficacy. The oral administration of some LAB strains like *L. acidophilus* exhibited anticancer activity in mice with breast cancer and increased the survival time in the *L. acidophilus*-supplemented group [13]. Also, the consumption of fermented milk products can reduce breast cancer incidence by influencing immune cells in mammary glands [17]. 

Although probiotics seem to have therapeutic potential in anticancer therapy, there are still some concerns. A growing body of evidence claims that oncological drugs alter gut microbiota, causing additional adverse effects [18]. Due to the antimicrobial activity of some routine therapeutics, they also could influence the viability of probiotics provided as supplements and therefore diminish their prohealth effects. This phenomenon has been highlighted recently by Li et al. for TAM, a drug widely applied in ER+ breast cancer therapy. Using the breast cancer xenograft mice model, the authors revealed that TAM is not neutral for the intestinal microbiome, which may result in increased inflammation [19]. The work by Diot et al. also demonstrates that supplementation with different microbial strains influences TAM action via the modulation of fatty acid metabolism in *C. elegans*. The authors suggested that a similar mechanism may modulate the therapeutic response to TAM in humans [20].

In recent years, postbiotics have gained considerable attention for their potential therapeutic properties. Compared to probiotics, they appear to be a reliable alternative, which eliminates the risk factors associated with applying living probiotic bacteria, such as the threat of bacteremia, in immunocompromised patients. Beyond the several established health-promoting benefits, an increasing amount of evidence suggests a novel dimension to postbiotics—their potential as agents with anticancer properties [21]. Based on in vitro studies, bacterial metabolites appear to have a potential role in both the prevention and treatment of many types of cancer [22,23]. Different LAB strains produce several metabolites presenting antineoplastic activity (Table 1). 

**Table 1:** Anticancer effect of postbiotics produced by lactic acid bacteria on various cancer cells.

LAB	Postbiotics	Anticancer Activity	Cancer Cell Line	Ref.
*L. plantarum*, *L. brevis*, *L. casei*	Butyric, propionic acid	Induction of apoptosis, decreased cell proliferation, ROS generation	Caco-2 (colorectal cancer) HeLa (cervical cancer)	[24]
*L. plantarum*	Bacteriocin	Decreased cell proliferation	MCF-7 (breast cancer) HT-29 (colorectal cancer)	[7]
*L. plantarum*	Bacteriocin (plantaricin)	Reduction of cancerogenic cell viability	E705 (colorectal cancer)	[25]
*L. plantarum*	Lipoteichoic acid	Anti-inflammatory effect	MDA-MB-231 (breast cancer)	[26]
*L. plantarum* *L. rhamnosus* *L. brevis*	Exopolysaccharides	Induction of apoptosis (Bax, Casp-3, Casp-9 overexpression)	HT-29 (colorectal cancer)	[27]
*L. rhamnosus* GG *L. casei*	Protein, polysaccharide	Anti-inflammatory effect; antimetastasis	HCT-116 c (colorectal cancer)	[28]
*L. rhamnosus* GG	Butyric acid	Inhibition of tumor growth, immunomodulation	Caco-2 (colorectal cancer)	[29]
*L. acidophilus*, *L. casei*	Polysaccharide fraction	Induction of apoptosis, antioxidative activity	HT-29 (colorectal cancer)	[30]

Among the various LAB species, *L. plantarum* 299v and *L. rhamnosus* GG are the most commonly investigated strains and are also widely used in the pharmaceutical and food industries. Analysis of the postbiotic composition of these strains revealed the presence of several bioactive compounds with antineoplastic potential, which can interact with the host’s immune system, modulate the tumor microenvironment, and exert a direct effect on cellular processes [31]. The products of bacterial metabolism with promising anticancer potential are short-chain fatty acids (SCFAs)—butyric, acetic, and propionic acids [29,32]. These compounds play the main role in the regulation of histone deacetylase activity (HDAC). The inhibition of HDAC modulates oxidative stress and reduces DNA damage in noncancerous epithelial cells by influencing DNA replication/repair systems and having an impact on apoptosis regulator genes [33]. Furthermore, some studies highlighted that SCFAs can induce apoptosis in tumor cells through the expression of p21 and Bcl-2, and the downregulation of cyclin B1, A, and D1 [34]. Moreover, in vitro studies have demonstrated that sodium butyrate can cause cell cycle arrest in colon cancer cells [35]. The next group of postbiotics with antineoplastic activity are bacteriocins synthesized by some of the LAB strains, e.g., nisin from *L. lactis* or plantaricin from *L. plantarum*. They exhibit selective cytotoxic effects on cancer cells and can distinguish them from normal ones, due to their higher affinity for the negatively charged membrane of neoplastic cells [36]. However, more precise cellular targets have also been recognized; for instance, plantaricin was observed to exert an antiproliferative effect on colon cancer cells by triggering EGFR [25]. Another type of anticancer LAB-derived macromolecules are exopolysaccharides, which can induce apoptosis in different types of cancer cells, including colon, liver, breast, and intestine neoplasms. Moreover, EPS have been reported to possess anti-inflammatory properties, reducing the inflammatory microenvironment that can promote cancer progression [37].

The majority of previous experiments investigating the anticancer activity of probiotics have focused on colon neoplasms, but there is an increasing number of findings revealing their potential in prophylaxis and the treatment of other types of cancer. Our results for postculture cell-free supernatants derived from *L. plantarum* and *L. rhamnosus* are consistent with previous findings showing the antineoplastic potential of probiotic metabolites (Figure 1) and demonstrating their ability to stimulate the apoptosis of breast cancer cells (Figure 2, Figure 3, Figure 5 and Figure 6) and influence their cell cycle and proliferation (Figure 7 and Figure 8). Furthermore, our findings demonstrated that LAB-PM exhibited selective cytotoxic activity against cancerous cells while exerting a supportive effect on the viability of non-neoplastic cells (WI-38 human cell line); (Figure 1b). Previous observations also demonstrated the nontoxic nature of postbiotics derived from LAB species on normal cells (noncancerous human breast MCF-10A cells, mice splenocytes, thymocytes, and human peripheral blood mononuclear cells) [7].

Based on the selective anticancer potential of postbiotics, Rad et al. considered them a novel approach for adjunctive therapy in patients with cancer [38]. To date, few studies seem to support this concept. Salemi et al. demonstrated the inhibitory activity of *L. rhamnosus* GG cell-free supernatant against human colon and melanoma cancer cell lines, with a nontoxic effect on fibroblast cells, although they did not observe cell death stimulation in cancer cells. They showed that a combination of *L. rhamnosus* postbiotics with 5-fluorouracil and irinotecan sensitizes cancer cells to chemotherapeutics [10].

Since *L. plantarum* PM and *L. rhamnosus* PM exhibited selective cytotoxicity against breast cancer cells, we evaluated their potential as adjunctive agents in combination with TAM. Tamoxifen, as a selective estrogen receptor modulator, acts as an antagonist to the ER in ER+ breast cancer. Binding TAM impairs estrogen-responsive pathways, resulting in cell cycle arrest in the G0/G1 phase and the promotion of apoptosis [39]. Since estrogen response is not an exclusive mechanism stimulating breast cancer proliferation and growth, therapeutic approaches potentially enhancing the antineoplastic effect of TAM are worth exploring. Our results confirmed the inhibitory effect of TAM, involving a block of the cell cycle in the G0/G1 phase and the almost complete downregulation of Ki-67 expression. The combination of TAM with LAB-derived postbiotics seemed to strengthen this effect (Figure 7 and Figure 8). Additionally, the proapoptotic activity of TAM was significantly enhanced by the addition of LAB-PM, even for the lower concentration of TAM (Figure 2). While LAB-PM can induce apoptosis per se, placing cells in the early phase of this process, the combination therapy accelerates this phenomenon, causing the cells to shift into the late apoptotic phase (Figure 3). A similar effect was observed with the newly tested candidate drug ARA12, a derivative of aziridine–hydrazide hydrazone (Figure 5 and Figure 6). The antineoplastic effect of ARA12 had been previously shown by our team in relation to glioblastoma cells [11]. When tested in MCF-7, it also appeared to be cytotoxic against breast cancer cells, decreasing their viability, inhibiting the cell cycle and proliferation, and inducing apoptosis (Figure 4, Figure 5, Figure 6, Figure 7 and Figure 8). A similar observation of ARA12 action was parallelly made by Ziółkowska et al. [40]. Importantly, at lower concentrations, the effect appeared to be selective to neoplastic cells, with significantly less harmful effects on noncancerous cells (Figure 4).

The addition of LAB-PM to ARA12 enhanced the cytotoxic effect on MCF-7, causing cell cycle arrest in the G0/G1 phase and a decrease in Ki-67 expression to an undetectable level, suggesting a block in proliferation (Figure 7 and Figure 8). The combined treatment also exerted a strong proapoptotic effect, generating above 60% of apoptotic cells, the majority of which were in the late phase of apoptosis (Figure 5 and Figure 6).

Although slight differences were detected between the efficiency of postbiotics derived from *L. plantarum* or *L. rhamnosus*, both showed antineoplastic activity against MCF-7 and the ability to support the therapeutic potential of applied chemical agents, with *L. plantarum* appearing to be more promising. One of the possible explanations is the fact that *L. planatrum* is recognized as a strain producing a variety of bacteriocins, known as plantaricins, that display selective anticancer activity [41,42,43]. Several plantaricins have been characterized in silico in relation to their therapeutic potential in lung cancer, followed by an in vitro cytotoxicity assay, the results of which confirmed their selective antitumor activity [44]. The ability of bacteriocins to improve the treatment efficacy of chemotherapeutic agents was demonstrated by Avand et al., who showed a synergistic effect of nisin and doxorubicin against breast cancer cells [45]. Upon initial observation, the proapoptotic potential of *L. plantarum* PM appeared to be comparable to that of chemical agents alone (TAM at 10 µM or ARA12), resulting in the generation of a similar number of apoptotic cells. However, a more comprehensive analysis revealed that cells treated exclusively with LAB-PM exhibited characteristics of early apoptosis, while the combined treatment had the effect of accelerating the apoptotic process.

The aforementioned studies, based on the use of LAB-derived cell-free supernatants and oncological drugs, have been tested on in vitro models, but studies on animal models have demonstrated that the in vivo application of LAB species or LAB-derived postbiotics can also act as antineoplastic agents of natural origin. The oral administration of *L. plantarum* to tumor-bearing mice has been shown to exert anticancer effects, as evidenced by the inhibition of adenocarcinoma growth and prolonged survival [46]. The coadministration of doxorubicin with *L. acidophilus*, *L. casei*, and vitamin D3 resulted in a reduction in tumor size in a mouse model of breast cancer and the alleviation of chemotherapy-related side effects [47]. 

The investigation by Hashemi-Khah et al. demonstrated the anticancer potential of *L. rhamnosus* cell-free supernatant, which was found to reduce tumor volume in mice affected with esophageal cancer to a comparable extent to that of synthetic chemotherapeutic 5-FU [48]. The delivery of *L. rhamnosus* GG cytoplasmic fraction using magnetic iron nanoparticles to breast cancerous tissue in a mouse model also demonstrated the antineoplastic effect of the probiotic metabolites in vivo. This was evidenced by a reduction in tumor size and volume and the enhancement of apoptosis [49].

There is also evidence of the adjuvant potential of bacteriocins from in vivo experiments. The results from the study of Rana et al. demonstrated enhanced therapeutic efficacy of 5-fluorouracil in combination with nisin against murine skin cancer [50,51]. Furthermore, Fu and Kapila’s case report suggested a supportive role of nisin in the chemoradiation treatment of metastatic oropharyngeal cancer in patients [52].

SCFAs have also been extensively studied for their supportive role in oncological therapy. This group of compounds has been shown to enhance the anticancer effects of several chemo- and immunotherapeutics, as well as the efficacy of radiotherapy. Importantly, they alleviate the unwanted toxicity of chemotherapeutics harmful to noncancerous tissue [53]. 

The potential of LAB and their metabolites as oncotherapeutic agents has also been investigated using probiotics isolated from regional food products. Nasiri et al. found evidence of a synergistic anticancer effect of tamoxifen and *L. brevis* derived from dairy products [54]. Our findings demonstrate improved cytotoxicity of this drug when combined with postbiotics derived from *L. plantarum* and *L. rhamnosus*, which is consistent with previous observations.

In addition to anticancer efficacy improvement, combining postbiotics with TAM has another added value. This is because TAM has been found to have undesired proinflammatory action, while several postbiotics have been shown to have anti-inflammatory effects [19,55].

As LAB species produce a range of bioactive molecules with anticancer properties, it is challenging to precisely determine the mechanisms underlying the synergy between postbiotics and oncological drugs. However, the existing data present a cohesive picture of their potential to support each other. The current study has shown that postbiotic metabolites from commonly used probiotics, namely *L. plantarum* and *L. rhamnosus*, can be considered adjunctive agents in breast cancer therapy conducted with tamoxifen. Additionally, they were found to support the activity of a new derivative of aziridine–hydrazide hydrazone, which is a promising candidate for anticancer treatment. This emphasizes the multipotential properties of postbiotics as natural adjunctive agents supporting anticancer therapy based on various synthetic drugs.

## 4. Materials and Methods

### 4.1. Bacterial Culture and Cell-Free Supernatant Preparation

The bacterial strains *Lactobacillus rhamnosus* GG and *Lactiplantibacillus plantarum* 299v were inoculated separately in a De Man, Rogosa, and Sharpe medium (MRS) at 30 °C overnight under limited oxygen conditions, with periodic shaking. After that, the bacterial suspension with an optical density (OD) of 0.8 measured at 600 nm (corresponding to 10^8^ CFU/mL) was obtained, and then the bacterial suspension was inoculated into DMEM (Gibco, Life Technologies Europe B.V., Bleiswijk, The Netherlands) without FBS and antibiotic solution overnight at 30 °C to eliminate the direct influence of MRS on eucaryotic cells in subsequent steps of analysis. Finally, the bacterial culture in DMEM was centrifuged for 20 min, 4000× *g*, the pellet was discarded, and the supernatant pH was adjusted to 7.4. Cell-free supernatants were filtered through a 0.22 μm membrane to remove remaining bacterial fragments and subsequently used for cell-based experiments, as a source of postbiotic metabolites. 

### 4.2. Cell Culture

The human ER-positive breast cancer cell line (MCF-7; ATCC No. HTB-22) and human fibroblast cells (WI-38; ATCC No. CCL-75) are established commercially available products originally purchased from the vendor (ATCC). The cells were cultured in Dulbecco’s modified Eagle’s medium—high glucose (DMEM-HG, Gibco, Life Technologies Europe B.V., Bleiswijk, The Netherlands). Both types of media were supplemented with 10% FBS (Gibco, Life Technologies Limited, Paisley, UK) and antibiotics, namely gentamicin (100 mg/mL) and streptomycin with penicillin (100 mg/mL) (Sigma-Aldrich, St. Louis, MO, USA), at 37 °C and 5% CO_2_ atmosphere.

### 4.3. Natural and Synthetic Compounds

Postbiotic metabolites were obtained as cell-free supernatants from the culture of LAB strains *L. plantarum* or *L. rhamnosus* (LAB-PM) according to the procedure described above. Tamoxifen (TAM) (T2859; Sigma-Aldrich) and ARA12, a new derivative of aziridine–hydrazide hydrazones with antineoplastic activity, were synthesized according to the protocol presented previously [11].

### 4.4. Cell Viability Assay

The cancer cell line MCF-7 and the normal cell line WI-38 were plated into 96-well microplates at 5 × 10^3^/well and incubated at 37 °C in a 5% CO_2_ for cytotoxicity assay. After 24 h, the cells were treated with the examined compounds, namely concentrations of LAB-PMs ranging from 0 to 50% (*v*/*v*) obtained from *L. plantarum* or *L. rhamnosus* and a range of concentrations of ARA12 (0, 25, 50, 100, 150, 200, 300, and 500 μg/mL) and TAM (0, 5, 10, 15, 20, 25, 30, and 35 µM). The selected concentration for natural agents (LAB-PM derived from *L. plantarum* or *L. rhamnsous*) was 30% (*v*/*v*), and for synthetic compounds, the selected concentrations were 20 µM for TAM and 25 μg/mL for ARA12; thus, the combination of LAB-PM with either 20 µM of TAM or 25 μg/mL of ARA12 was used. Then, after 72 h, the PrestoBlue™ Cell Viability Reagent (Thermo Scientific, Rockford, IL, USA) was added to each well, and the plates were incubated for 2 h at 37 °C in 5% CO_2_. The fluorescence signal was recorded using a microplate reader (Glomax Multi Detection System; Promega, Madison, WI, USA).

### 4.5. Annexin V/PI Staining for Cell Death Assay

Apoptosis in both cell lines was detected by an Annexin V Apoptosis Detection Kit (BD Biosciences, Erembodegem, Belgium). The MCF-7 cells were plated into 6-well plates at 1 × 10^5^/well and incubated at 37 °C in a 5% CO_2_. After 24 h, the cells were treated with the tested drugs alone—TAM (10 µM and 20 µM) and ARA12 (25 μg/mL)—or in combination with bacterial supernatants from *L. plantarum* and *L. rhamnosus* in a concentration of 30% (*v*/*v*) for 72 h. After incubation, cell pellets were suspended in PBS, centrifuged, and stained with annexin V-FITC/propidium iodide (PI) at room temperature in the dark for 15 min, according to the manufacturer’s instructions. A flow cytometry device (CytoFLEX Beckman Coulter, Brea, CA, USA) was used for cellular analysis. 

### 4.6. Cell Cycle Analysis

The MCF-7 cells were plated into 6-well plates at 1 × 10^5^/well and incubated at 37 °C in 5% CO_2_. After 24 h, the cells were treated with tested drugs alone—TAM (10 µM and 20 µM) and ARA12 (25 μg/mL)—or in a combination of bacterial supernatants from *L. plantarum* and *L. rhamnosus* (30% *v*/*v*) and the tested drugs for 48 h. After that, cells were harvested and suspended in 75% ice-cold ethanol and stored at −20 °C. After the next 24 h, the cells were processed with propidium iodide (PI)-staining solution (PI/RNase Staining Buffer, BD Biosciences, Erembodegem, Belgium) and kept at room temperature for 15 min. Cell cycle distribution was measured using flow cytometry (CytoFLEX Beckman Coulter, Brea, CA, USA).

### 4.7. Ki-67 Immunofluorescence Detection

The MCF-7 cells were plated onto coverslips at 2.5 × 10^4^/well and treated with tested drugs alone—TAM (20 µM) and ARA12 (25 μg/mL)—or in a combination with *L. plantarum* PM or *L. rhamnosus* PM in a concentration of 30% (*v*/*v*). After 48 h treatment, cells were fixed with 4% paraformaldehyde for 15 min. Then, cell membranes were permeabilized with 0.1% Triton X-100 for 10 min, following incubation with 2% goat serum in PBS for 1 h to block nonspecific binding sites. Afterward, the cells were incubated for 1 h with the primary antibody (1:250, mouse monoclonal anti-Ki-67 antibody, P6834, Sigma-Aldrich, St. Louis, MO, USA). For microscopy visualization, the cells were incubated with a secondary antibody conjugated with a fluorochrome (1:500, goat anti-mouse Alexa-Fluor568, Thermo Scientific, Rockford, IL, USA) for 1 h in the dark. Slides were mounted with ProLong Glass Antifade Mountant (Thermo Scientific, Rockford, IL, USA). Immunofluorescence results were examined using a fluorescence microscope and subjected to semiquantitative analysis based on the assessment of cells positive for Ki-67 expression (for each sample, signals from at least 100 cells were counted from 3 separate microphotographs performed with the same exposure time).

### 4.8. Statistical Analysis

All the experiments were repeated at least three times, and the data were expressed as the means ± SD. Comparisons between the two groups were performed using Student’s *t*-test, and differences among three or more groups were determined using one-way analysis of variance (ANOVA) followed by the Holms–Sidak post hoc test. Multiplicity adjusted value of *p* < 0.05 was considered statistically significant. 

## Data Availability

All data generated or analyzed during this study are included in this article.
